# SEX- AND AGE-SPECIFIC METABOLIC EFFECTS OF EARLY-LIFE RAPAMYCIN TREATMENT IN UM-HET3 MICE

**DOI:** 10.1093/geroni/igae098.3713

**Published:** 2024-12-31

**Authors:** Yun Zhu, Robert Stockwell, David Medina, Rong Yuan, Andrzej Bartke

**Affiliations:** Southern Illinois University School of Medicine, Springfield, Illinois, United States; Southern Illinois university Shool of Medicine, Springfield, Illinois, United States; Southern Illinois University School of Medicine, Springfield, Illinois, United States; Southern Illinois university Shool of Medicine, Springfield, Illinois, United States; Southern Illinois University School of Medicine, Springfield, Illinois, United States

## Abstract

UM-HET3 mice were treated with rapamycin (4 mg/kg, i.p., 3 times/week) from 15 to 56 days of age. At day 15, no significant differences in body weight (BW) between the treatment and control groups within each sex. During the treatment, males in the treated group consistently had lower BW than controls (pairwise-t test, P < 0.01), while no differences were seen in females. Interestingly, the treatment significantly reduced the relative weights of fat pads (visceral, subcutaneous, and brown fat) in males, with a suggestive reduction in the relative pancreas weight and significant increases in heart, liver, and kidney relative weights. No significant organ weight changes were observed in females. At 2 months, rapamycin significantly raised fasting glucose in males (t test, P = 0.0002) but not in females. By 6 months, fasting glucose levels were significantly reduced in treated mice compared to controls, with a 26.7% reduction in males and 15.7% in females (t test, P = 0.0001 and 0.041, respectively). Glucose tolerance, assessed by the area under the curve (AUC) of relative glucose levels, was significantly increased by rapamycin in both sexes at 2 and 6 months (t test, P < 0.05), except in 6-month-old males. No significant effects or interactions were found in the insulin tolerance test. Indirect calorimetry showed no significant changes in heat production, but a suggestive increase in RQ was observed in treated males. These findings suggest that early-life rapamycin treatment may have long-term effects on metabolic parameters, and the effects are sex- and age-specific.

